# Secured closed system drainage of a retrograde ureteric catheter

**DOI:** 10.1308/003588413X13511609957056k

**Published:** 2013-01

**Authors:** S Pathak

**Affiliations:** Doncaster and Bassetlaw Hospitals NHS Foundation Trust, UK

## Background

Retrograde ureteropyelography plays a vital role in imaging of the upper tract, particularly in aiding the diagnosis of upper tract urothelial cancers.^1^ Retrograde studies in theatre are often of inferior quality compared with those performed in the radiology department. Retrograde ureteric catheter placement in theatre is therefore followed by formal retrograde studies in the radiology department. However, it is not uncommon for the retrograde catheter to become displaced in the journey from theatre to the radiology department. We describe a secured, closed system drainage of the ureteric catheter into the urine drainage bag via the urethral catheter.

## Technique

**Figure 1:**
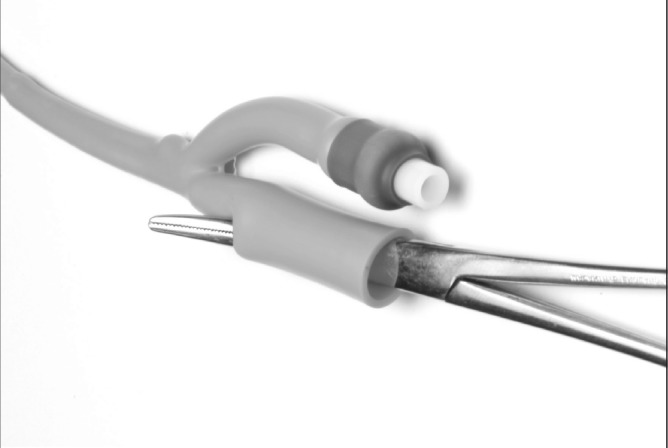
A hole is punctured into the distal end of the urethral catheter.

**Figure 2:**
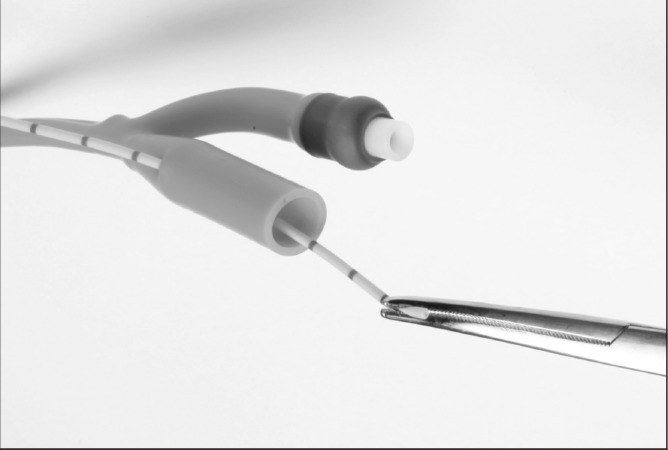
The distal end of the ureteric catheter is grasped and pulled through into the urethral catheter.

**Figure 3:**
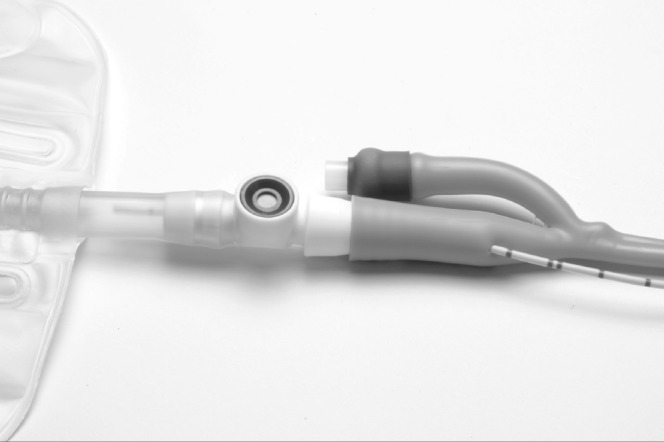
The urinary drainage bag is connected to the urethral catheter.

Under radiological imaging, a retrograde ureteric catheter is placed in the upper ureter/renal pelvis. The urethral catheter is placed into the urinary bladder, the balloon inflated and gently pulled down, taking care not to displace the ureteric catheter. The tips of the small artery forceps are used to puncture a hole into the distal end of the urethral catheter (Fig 1). The distal end of the ureteric catheter is grasped with the tips of the forceps and gently pulled through into the urethral catheter (Fig 2). The urinary drainage bag is connected to the urethral catheter, allowing the ureteric catheter to also drain into the bag (Fig 3). 

## Discussion

This technique allows the ureteric catheter to be secured firmly until the formal retrograde studies are performed in the radiology department. Furthermore, the closed system minimises the risks of introducing infection into the urinary tract.

## References

[1] Browne RF , Meehan CP , Colville J *et al* Transitional cell carcinoma of the upper urinary tract: spectrum of imaging findings. Radiographics 2005; 25: 1,609–1,627 10.1148/rg.256045517 16284138

